# Evaluation of Beauvericin’s activity and mode of action against all life stages of *L. tropica* for cutaneous Leishmaniasis therapy

**DOI:** 10.3389/fcimb.2025.1599766

**Published:** 2025-06-10

**Authors:** Lynn Al Samra, Mohamad El Nahas, Ilham Mneimneh, Aia Sinno, Sima Tokajian, Kelven Rahy, Sergio Thoumi, Lazo Ali, Wael Yammine, Charbel Al Khoury

**Affiliations:** ^1^ Department of Natural Sciences, School of Arts and Sciences, Lebanese American University, Chouran, Beirut, Lebanon; ^2^ Department of Computer Science and Mathematics, Lebanese American University, Beirut, Lebanon; ^3^ Department of Natural Sciences, School of Arts and Sciences, Lebanese American University, Byblos, Lebanon; ^4^ Gilbert and Rose-Marie Chagoury School of Medicine, Lebanese American University, Byblos, Lebanon; ^5^ Department of Plant Protection, Faculty of Agricultural & Veterinary Sciences, Lebanese University, Dekwaneh, Beirut, Lebanon

**Keywords:** Leishmaniasis, *Leishmania tropica*, Beauvericin, drug resistance, RNA-Seq, *Galleria mellonella* model

## Abstract

**Background:**

Leishmaniasis, particularly its cutaneous form caused by *Leishmania tropica*, remains a significant global health concern due to the limitations of current treatments, including drug resistance, toxicity, and inconsistent efficacy. This study investigates the potential of Beauvericin (BEA), a fungal secondary metabolite, as an alternative antileishmanial agent.

**Objectives:**

This study investigates the potential of Beauvericin (BEA), a fungal secondary metabolite, as an alternative antileishmanial agent.

**Methods:**

We assessed the efficacy of BEA against different developmental stages of *L. tropica* using *in vitro* assays and an *in vivo Galleria mellonella* infection model. The ability of *L. tropica* to develop resistance to BEA and its effects on the parasite’s gene expression profile were also examined.

**Results:**

BEA exhibited potent antileishmanial activity with equipotency across both promastigote and amastigote stages of *L. tropica*, with IC_50_ values of 0.25 µM and 0.27 µM, respectively, significantly lower than those of miltefosine. Mechanistically, BEA acts as a calcium ionophore, inducing a marked increase in intracellular calcium levels, which serves as the primary cytotoxic event. Transcriptomic profiling further revealed that BEA-induced calcium dysregulation triggers secondary cellular responses involving calcium homeostasis, lipid metabolism, and stress response, contributing to its multifaceted mechanism of action. The *G. mellonella* model demonstrated that BEA significantly reduced parasite burden, improved survival rates. Notably, BEA showed a slower rate of resistance development compared to ML, indicating its potential as a more sustainable treatment option.

**Conclusions:**

BEA is a promising candidate for antileishmanial therapy, demonstrating superior efficacy, a broad mechanism of action, and a favorable resistance profile compared to ML. Further investigations in mammalian models are warranted to validate BEA’s potential as a novel, cost-effective treatment for leishmaniasis.

## 1 Introduction

Leishmaniasis is a vector-borne disease caused by the invasion of a flagellated protozoan parasite from the genus *Leishmania* (Akhoundi et al., 2016). *Leishmania* exhibits a dimorphic life cycle (Wheeler et al., 2011). The transmission cycle begins when the female sandfly takes a blood meal and regurgitates the promastigotes into the host’s bloodstream. These flagellated promastigotes are then phagocytosed by macrophages (Naderer and McConville, 2008). Once inside the macrophages, promastigotes rapidly transform into non-flagellated intracellular amastigotes and begin replicating through binary fission (Naderer and McConville, 2008). This replication process eventually leads to macrophage rupture, allowing the amastigotes to spread to neighboring cells (Naderer and McConville, 2008). During subsequent blood meals, sandflies ingest these infected phagocytic cells, facilitating the transmission of amastigotes (Dostálová and Volf, 2012). Once in the sandfly’s midgut, the amastigotes convert back into procyclic promastigotes, migrate to the proboscis, and differentiate into metacyclic promastigotes, completing the cycle (Dostálová and Volf, 2012). Leishmaniasis presents substantial challenges in terms of management and control, particularly in regions with high population density, poverty, and inadequate sanitation (Grifferty et al., 2021). Annually, over one million cases are reported, putting more than one billion people at risk in endemic areas with more than 20 species of *Leishmania* responsible for three major clinical manifestations (World Health Organization, 2024). Cutaneous leishmaniasis (CL), the most common form, is caused by *L. major* and *L. tropica*, leading to potentially scarring skin lesions (World Health Organization, 2024). The Mediterranean region, with Syria having the highest prevalence, accounts for 57% of CL cases, driven by conflict and poor conditions in refugee camps, spreading the disease to Turkey, Iraq, and specifically Lebanon (Bizri et al., 2021). In addition to its prevalence among civilian populations, *L. tropica* has also emerged as a significant concern in military settings, with documented outbreaks among deployed personnel in endemic regions (Niba Rawlings et al., 2025).

Pharmaceutical companies have shown limited interest in developing antileishmanial drugs due to the perceived high-risk nature of such investments (de Vries and Schallig, 2022). Current treatments, such as pentavalent antimonials, miltefosine (ML), and amphotericin B, face significant drawbacks including toxicity, limited availability, and drug resistance (Zhang et al., 2025; Douanne et al., 2022). These challenges, along with the lack of effective vaccines, highlight the need for new, accessible treatments for CL.

Given their widespread availability, cost-effectiveness, and biological diversity, natural products have emerged as compelling candidates in the search for new antileishmanial therapies (Fatima et al., 2016). The potential of these compounds is underscored by numerous studies that have successfully harnessed the bioactive properties of natural sources, including microorganisms, to develop synthetic derivatives with potent activity against *Leishmania* species (Orosco et al., 2024). Among these natural products, Beauvericin (BEA), a secondary metabolite from *Beauveria bassiana*, stands out due to its broad spectrum of biological activities (Al Khoury et al., 2024a). Interestingly, previous studies reported antiparasitic activities of BEA against *Sarcoptes scabiei* (Al Khoury et al., 2020), *Trypanosoma cruzi* (Campos et al., 2015), and promastigotes of *L. braziliensis* (Nascimento et al., 2012). The diverse biological activities of BEA indicate its potential for significant biomedical applications, particularly in the development of new treatments for leishmaniasis. Nevertheless, the escalating issue of drug resistance in *Leishmania* spp. further complicates treatment efforts. The emergence of resistance is largely attributed to the parasite’s genomic plasticity, which facilitates rapid adaptation to pharmacological pressures (Laffitte et al., 2016). These adaptive responses are particularly concerning in endemic regions where prolonged drug exposure is common, leading to a pressing need for novel therapies that are less prone to resistance development. The potential for BEA to circumvent resistance mechanisms has been previously reported, suggesting that it could serve as a promising candidate for treating highly adaptable and resilient organisms such as *Leishmania* (Nascimento et al., 2012). To comprehensively evaluate BEA’s potential in overcoming these challenges, a deeper understanding of its molecular mechanisms is crucial. Advancing our understanding of BEA’s antileishmanial properties necessitates a detailed exploration of its molecular mechanism of action. Recent advancements in transcriptomic technologies, notably RNA sequencing (RNA-Seq), provide a powerful platform for elucidating the genomic and transcriptomic responses of *Leishmania* to drug treatment (Salloum et al., 2021). RNA-Seq offers unparalleled resolution in detecting differential gene expression, enabling the identification of disrupted metabolic pathways, stress response mechanisms, and potential secondary targets of BEA. However, to translate these molecular insights into practical treatments, rigorous *in vivo* validation is essential. *In vivo* models remain critical for validating the translational potential of *in vitro* findings. While mammalian models are traditionally used in infectious disease research, alternative models such as *Galleria mellonella* (the greater wax moth larva) have gained recognition for their ethical advantages, cost-effectiveness, and applicability to high-throughput screening (Tomiotto-Pellissier et al., 2016). *G. mellonella* has been successfully employed in the study of host-pathogen interactions, offering a simplified yet relevant model for assessing the immunomodulatory effects of potential therapies. Its utility in evaluating BEA’s efficacy against *Leishmania* infections could provide valuable preclinical data, supporting the progression of BEA into clinical trials. Taken together, this study aims to assess the efficacy of BEA across all developmental stages of *L. tropica* and its interactions with host macrophage-like cells; to investigate the potential for resistance acquisition by *L. tropica* in response to BEA treatment; to utilize RNA-Seq technology to unravel the molecular mechanisms underpinning BEA’s antileishmanial activity; and to validate these findings using the *G. mellonella* model. This comprehensive approach aims to provide a robust evaluation of BEA’s therapeutic potential.

## 2 Materials and methods

The differentially expressed gene lists in promastigotes and intracellular amastigotes of *L. tropica* are listed in the Supporting Information.

### 2.1 Molecules to be evaluated

BEA 97% and ML 98% were purchased from Sigma-Aldrich (USA). Both compounds were stored according to the manufacturers’ guidelines until use.

### 2.2 Parasite culture and maintenance

The *L. tropica* LT2 strain (MHOM/LB/2015/IK) was isolated from punch biopsies in 2014 at the American University of Beirut Medical Centre (AUBMC), as described by Salloum et al. (2020). The promastigotes were cultured in RPMI-1640 medium (Sigma) supplemented with 1% penicillin-streptomycin (Biowest) and 20% heat-inactivated fetal bovine serum (FBS) (Sigma) and maintained at 25°C in a 5% CO_2_ incubator. THP-1 cells were cultured in RPMI-1640 (Sigma) with 1% penicillin-streptomycin (Biowest) and 20% FBS (Sigma) and incubated at 37°C with 5% CO_2_. Non-adherent THP-1 cells were seeded in 6-well plates and differentiated into macrophage-like cells using 25 ng/mL Phorbol 12-Myristate 7-Acetate (PMA) (Fisher) overnight. The cells were then washed with PBS (Sigma) to remove non-adherent cells and activated with 1 ng/mL lipopolysaccharide (LPS) (Invivogen) derived from *Escherichia coli* 0111 for four hours to stimulate an immune response. Metacyclic promastigotes were introduced at a 10:1 ratio to the macrophage-like cells to obtain intracellular amastigotes, and the cultures were incubated for 48 hours at 37°C with 5% CO_2_. Extracellular promastigotes were removed with two PBS washes.

### 2.3 Efficacy of BEA against different developmental stages of *L. tropica* and macrophage-like cells

BEA was tested at concentrations of 0.01, 0.05, 0.1, 0.5, 1, 2, 5, 7, and 10 μM over 72 hours on one million macrophage-like cells, *L. tropica* promastigotes, intracellular amastigotes, and axenic amastigotes. In addition, ML was used as a positive control due to its previously demonstrated leishmanicidal activity. Cell viability was assessed using reverse transcription-quantitative polymerase chain reaction (RT-qPCR) as described by Al Khoury et al. (2022a). In brief, total RNA was extracted from treated and untreated control cells using the RNeasy Mini Kit (Qiagen, Hilden, Germany), following the manufacturer’s instructions. Subsequently, 2 µg of RNA was reverse transcribed into cDNA using the RevertAid First Strand cDNA Synthesis Kit (#K1622-Thermo Scientific). SYBR^®^ Green 2× (Sigma Aldrich) was employed to quantify the expression of kDNA and GAPDH, serving as marker genes for the parasites and macrophage-like cells, respectively. Double-stranded DNA (dsDNA) purified from conventional PCR reactions was used as a standard. The input target sequence was quantified by plotting the Ct values of cells against the standard curves. Experiments were conducted in triplicate, with each experiment repeated three times. The half-maximal inhibitory concentration (IC_50_) for BEA was calculated for each experiment (**Table 1**).

**Table 1:** IC_50_ values of BEA and ML against *L. tropica* Promastigotes, Amastigotes, and macrophage-like cells, along with calculated selectivity indices (mean ± S.E.).

Cell type	IC50 µM (mean ± SE)	
	Beauvericin	Miltefosine
Promastigotes	0.25 ± 0.002	1.50 ± 0.13
Amastigotes	0.27 ± 0.002	2.33 ± 0.14
Macrophage-like cells	2.618 ± 0.01	62.33 ± 1.37
	Selectivity index* (mean ± SE)	
	9.69 ± 0.01	26.75 ± 0.34

*Selectivity Index was calculated as the ratio of CC_50_ (Macrophage-like cells) to IC_50_ (Amastigotes).

### 2.4 Resistance selection assay

The resistance selection assay was performed as previously described (Al Khoury et al., 2024b). Briefly, the drug-susceptible *L. tropica* strain (LS-LT2) was initially maintained without drug exposure. Subsequently, the strain was subjected to increasing concentrations of BEA (0 to 50 µM) across rounds of selection to impose selective pressure, aiming to reduce the parasite load by 50%. The IC_50_ and BEA efficacy were assessed at intervals of five rounds of selection across different developmental stages of *L. tropica*. After 15 rounds of selection, strains resistant to BEA (BEAR-LT2) and ML (MLR-LT2) were identified. Resistance ratios (RR) were calculated by dividing the IC_50_ of each round of selection by that of the original LS-LT2 strain. Promastigotes were cultured in RPMI-1640 medium with 5% FBS at a density of 10^6^ cells/mL. The population was exposed to increasing BEA concentrations, targeting a 50% reduction in the initial population. Cells were then transferred to fresh media and incubated for an additional 72 hours to restore the population to 10^6^ cells/mL before being re-exposed to BEA. Intracellular amastigotes were seeded in 96-well plates and also exposed to the concentration of BEA required to kill 50% of the parasites. After incubation, the plates were washed three times with PBS. For parasite rescue and transformation, PBS was replaced with 20 µl of RPMI-1640 supplemented with 5% sodium dodecyl sulfate (SDS) (BIO-RAD 1610301) (Jain et al., 2012). After 30 seconds of shaking, 180 µl of RPMI-1640 with 10% FBS was added to each well. This step facilitated controlled lysis of infected macrophages with minimal loss of viability in the rescued parasites. Plates were then incubated at 27°C for 48 hours to allow complete back-transformation of intracellular amastigotes to promastigotes. Upon detection, promastigotes were expanded in a 25 mL flask. The resulting metacyclic promastigotes were used to infect macrophage-like cells for the next round, where intracellular amastigotes were again exposed to the drug. These experiments were conducted in triplicate and repeated three times.

### 2.5 Measurement of intracellular calcium in *L. tropica*

*L. tropica* promastigotes were cultured as indicated above, then harvested, washed with PBS, and resuspended to 1×10^8^ cells/ml. For calcium measurement, cells were treated with the IC_50_ value of BEA, an equal concentration of A23187 (positive control), a known calcium ionophore, or left untreated (blank), then incubated at 25°C for 4 hours. Post-incubation, cells were washed and resuspended in Ca NW PLUS solution (DiscoverX HitHunter™ Calcium NoWashPLUS Assay Kit), and 100 μl was transferred to black 96-well plates. Fluorescence was measured using a Perkin Elmer Multilabel Plate Reader (excitation 485 nm, emission 510 nm). This experiment was repeated three times in triplicates.

### 2.6 RNA-Seq and transcriptome analysis

In light of the marked increase in intracellular calcium levels induced by BEA treatment, we proceeded to investigate the broader transcriptomic consequences of this ionic dysregulation in *L. tropica* through high-resolution RNA sequencing. Both promastigote and intracellular amastigote stages were exposed to BEA at their respective IC_50_ concentrations for 4 hours. These conditions were selected to induce early-stage molecular perturbations without causing extensive cell death or transcriptomic degradation. Cell viability and morphological integrity were assessed via RT-qPCR prior to RNA extraction, ensuring that parasites remained intact and transcriptionally active at the time of sampling. Next, total RNA was extracted from BEA-treated and untreated promastigotes, as well as intracellular amastigotes, using the RNeasy Mini Kit (Qiagen, Hilden, Germany), following the manufacturer’s instructions. To eliminate potential genomic DNA contamination, RNA samples were treated with DNase I (Fermentas, Catalog # EN0521) for 30 minutes at 37°C. Purification was performed using RNeasy columns with 70% ethanol. RNA concentration and purity were assessed using the Bioanalyzer (Agilent 2100; BioRad) and Nanodrop 1000 (NanoDrop Technologies, Wilmington, DE, USA). RNA integrity was confirmed by electrophoresis on a 1.5% agarose gel. Paired-end RNA sequencing was conducted at Macrogen (Korea) on the HiSeq 4000 platform using the TruSeq Stranded Total RNA LT Sample Prep Kit (Gold). Poly-A enrichment was used to generate 101 bp strand-specific paired-end reads. Data quality was enhanced using Trimmomatic (v0.39) for adapter removal and low-quality read trimming, based on (Conesa et al., 2016). Read quality was assessed with FastQC (v0.11.9) before and after trimming. Reads were trimmed using a four-base sliding window, discarding those with an average quality per base below 15 and reads shorter than 36 bases. The %Q30 score was 98%, indicating high data accuracy. RNA-Seq data were analyzed using the Tuxedo pipeline, aligning reads to the *L. tropica* LT2 strain (MHOM/LB/2017/IK) genome. TopHat2 v2.1.1 was used for alignment, and unspliced reads were mapped using Bowtie v1.3.1 (Trapnell et al., 2009; Langmead, 2010). Unmapped reads were fragmented and aligned to identify splice junctions. The transcriptome was reconstructed with Cufflinks v2.2.1 and merged using Cuffmerge v2.2.1 (Ghosh and Chan, 2016). Differential gene expression was analyzed using Cuffdiff v2.2.1 (Ghosh and Chan, 2016), identifying differentially expressed genes (DEGs) between BEA-treated cells and controls. Genes were considered differentially expressed if they met a false discovery rate (FDR)-adjusted p-value threshold of < 0.05 and an absolute log_2_ fold change ≥ 0.5, as determined by Cuffdiff v2.2.1. This experiment was repeated three times in triplicates. Over-Representation Analysis (ORA) was subsequently performed to systematically identify GO terms significantly enriched among the DEGs. Using the clusterProfiler package (v4.6.2) in R, DEGs were mapped to GO categories (Biological Process, Molecular Function, and Cellular Component). To generate biologically coherent gene sets for downstream analysis, enriched GO terms were carefully reviewed and grouped according to shared functional themes. Genes associated with related GO terms were consolidated into unified sets representing key cellular processes such as transmembrane transport, kinase signaling, oxidative stress response, and calcium-dependent regulation (**Supplementary Note 1**). Gene Set Enrichment Analysis (GSEA) was conducted using RStudio (version 4.3.0) to classify genes according to their expression levels and identify enriched gene sets within the transcriptome following BEA exposure (Hung et al., 2012). Gene sets were constructed by integrating DEGs with Gene Ontology (GO) annotations, enabling the identification of overrepresented GO terms. These terms were used to group genes with similar functions, forming biologically relevant gene sets (Subramanian et al., 2005). This approach enabled the detection of gene sets with significant differential expression in response to BEA treatment in both promastigotes and intracellular amastigotes. Consequently, nine enriched gene sets were identified, which are detailed in **Table 1**.

### 2.7 *Galleria mellonella* infection model

*G. mellonella* larvae were used as an insect model to evaluate the therapeutic efficacy of drugs against leishmaniasis, based on recent findings supporting its utility in studying *Leishmania* spp (under review). Larvae were maintained on a wax and honey diet and kept in an oxygenated incubator at 28°C. Uniformity was ensured by selecting larvae with consistent pale pigmentation and an average weight of 300 mg. Hemolymph was collected by puncturing a larval proleg with a Hamilton syringe, yielding an average of 27.4 µl per larva. The hemolymph was chilled, mixed with 500 µL of insect physiological saline (IPS), centrifuged, washed, and resuspended in 1000 µL of IPS. Hemocyte counts were determined using a Neubauer chamber. Infection was established by sterilizing the larvae’s abdominal region with 70% ethanol and introducing *L. tropica* metacyclic promastigotes at a 1:10 host cell (hemocyte)-to-parasite ratio. Treatment was administered by injecting 10 µL of compound solution into the last left proleg using a sterile Hamilton syringe. Test compounds were initially dissolved in DMSO to prepare concentrated stock solutions, which were then diluted in IPS to achieve the final working concentrations. The final injection mixtures contained ≤10% DMSO to ensure solubility while avoiding vehicle-associated toxicity in the larvae. Administration of the vehicle alone had no observable effect on larval survival, behavior, or melanization compared to untreated controls. Based on allometric scaling, larvae were treated with either BEA (0.1 mg/Kg) or ML (2.5 mg/Kg). A pilot study was conducted where a range of BEA concentrations (0.01, 0.025, 0.05, 0.1, 0.2, 0.3, and 0.5 mg/kg) was tested, and 0.1 mg/kg was selected for further experiments due to its significant efficacy with no side effects. Higher concentrations showed no added benefits, confirming 0.2 mg/kg as the optimal dose. *L. tropica* proliferation within the hemolymph was monitored over 14 days post-infection. For quantification, hemocoel contents (including hemocytes and *Leishmania*) were pooled from groups of three infected larvae and analyzed by RT-qPCR. This assay was performed in duplicate, with the experiment repeated 10 times. Larval behavior, including mortality and melanization, was monitored for 14 days. Death was defined by immobility and lack of response to stimuli, while melanization was indicated by the darkening of the larvae’s surface. The study included four groups: RPMI-injected larvae (placebo), promastigote-infected larvae (PIL), PIL treated with ML, and PIL treated with BEA. Each group underwent 10 independent experiments with 10 larvae per group.

### 2.8 Statistical analysis

IC_50_ values and their standard errors (mean ± SE) were calculated using probit regression in SPSS Statistics for Windows, version 25.0 (IBM Corp., Armonk, NY). The therapeutic index of the BEA against different developmental stages of *L. tropica* was analyzed using two-way ANOVA. To assess the cytotoxicity of BEA and ML against macrophage-like host cells, an independent samples t-test was performed on the IC_50_ values obtained for each drug. For each developmental stage, resistance levels to BEA and ML were compared at each round of selection (selection rounds 5, 10, and 15). Within each round, resistance values between the two drugs were analyzed using independent two-sample t-tests. To control for the risk of Type I error associated with multiple comparisons, Bonferroni correction was applied, and statistical significance was determined using a threshold of p < 0.01. Differentially expressed gene sets were identified through GSEA performed with RStudio version 4.3.0. Genes were considered significantly differentially expressed if they exhibited an FDR-adjusted p-value < 0.05, as determined by Cuffdiff v2.2.1. Genes with a log_2_ fold change > 0 and FDR < 0.05 were classified as upregulated, while those with a log_2_ fold change < 0 and FDR < 0.05 were classified as downregulated. Genes not meeting this statistical threshold were not described as transcriptionally modulated (i.e., upregulated or downregulated) in the manuscript. The intracellular calcium concentration data was expressed as mean ± standard deviation (SD), and statistical significance was determined using one-way ANOVA followed by Tukey’s *post-hoc* test, with significance set at P ≤ 0.05. The *in vivo* efficacy of BEA was assessed by calculating the Area Under the Curve (AUC) for each *G. mellonella* larva using the trapezoidal rule, integrating the log concentration of *L. tropica* over the 14-day period to summarize the drug’s overall therapeutic effect. Significant differences in AUC between groups were tested using one-way ANOVA, followed by Tukey’s Honest Significant Difference (HSD) test to pinpoint specific differences. The *in vivo* therapeutic index, represented by survival and melanization of *G. mellonella* larvae, was analyzed using Kaplan-Meier curves. Statistical differences between experiment groups and untreated larvae were determined with a log-rank test, expressed through chi-square results and P values (df = 1). A P value of ≤ 0.05 is considered significant.

## 3 Results

### 3.1 Efficacy of BEA against *L. tropica* as well as macrophage-like cells

The efficacy of BEA and ML against *L. tropica* promastigotes and amastigotes was assessed by determining the IC_50_ values for each drug across both developmental stages. Notably, BEA’s IC_50_ values were consistent between promastigotes (IC_50_ = 0.25 ± 0.002 µM; mean ± SE) and amastigotes (IC_50_ = 0.27 ± 0.002 µM), with no statistically significant difference observed, suggesting equal potency across these developmental stages. In contrast, ML exhibited a significant reduction in efficacy against the clinically relevant amastigotes (IC_50_ = 2.33 ± 0.14 µM) compared to promastigotes (IC_50_ = 1.50 ± 0.13 µM), highlighting a stage-dependent decrease in susceptibility. A two-way ANOVA revealed significant main effects of both drug type (F(1,8)=765.85, p<0.05) and developmental stage (F(1,8)=49.59, p<0.05) on IC_50_ values, as well as a significant interaction between these factors (F(1,8)=46.57, p<0.05). *Post-hoc* analysis using Tukey’s HSD indicated that BEA had significantly lower IC_50_ values than ML across both stages, demonstrating superior efficacy (**Table 1**). These results underscore the potential of BEA as a more potent and stage-independent antileishmanial agent compared to the current standard, ML. The cytotoxicity of BEA and ML against macrophage-like cells was also evaluated by determining their respective IC_50_ values. BEA showed a mean CC_50_ of 2.61 ± 0.01 µM, indicating relatively high cytotoxicity. In contrast, ML exhibited a significantly higher mean CC_50_ of 62.33 ± 1.37 µM. An independent samples t-test confirmed that the difference in cytotoxicity between BEA and ML is statistically significant (t(4)=−76.54, p<0.05).

### 3.2 Resistance acquisition of promastigotes and intracellular amastigotes

**Figure 1:**
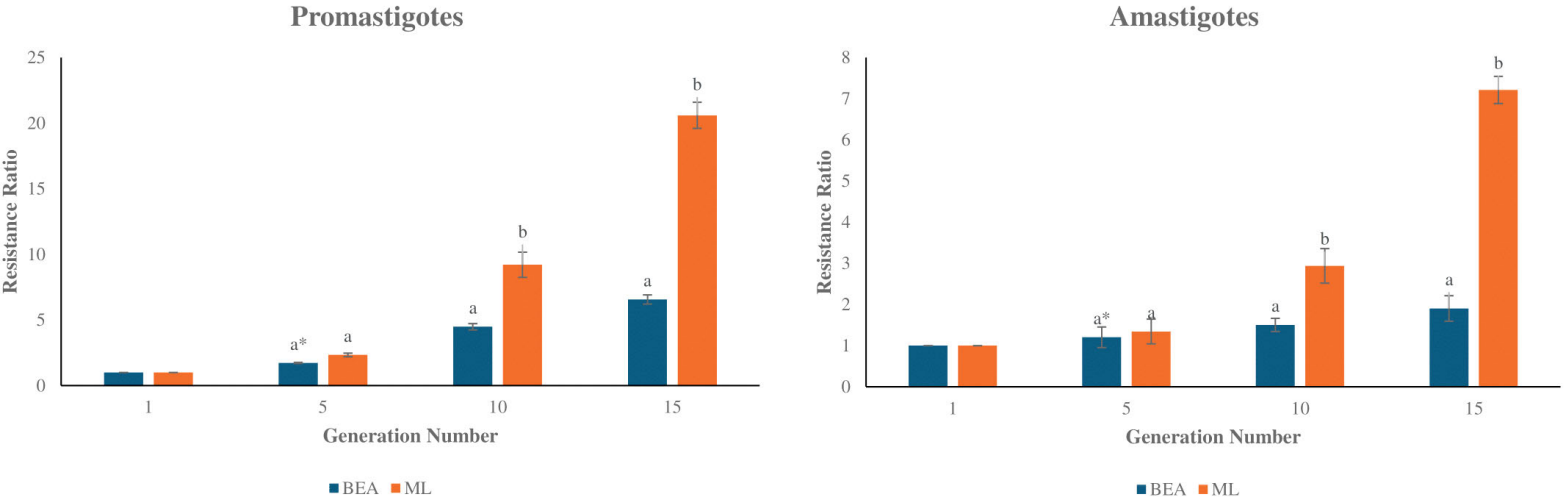
Development of resistance to BEA and ML across rounds of selection in *L. tropica* Promastigotes and Amastigotes. Bar plots depict the mean resistance ratios (± S.E.) of Promastigotes (left panel) and Amastigotes (right panel) to BEA and ML at rounds 1, 5, 10, and 15. Resistance was measured as the fold increase relative to the parental population (round 1). Independent two-sample t-tests were used to compare resistance between BEA and ML at each round of selection, with p-values adjusted using the Bonferroni correction to account for multiple comparisons. * Bars with different letters indicate significant differences (p < 0.01).

The present study highlights significant differences in the development of drug resistance in *L. tropica* based on the type of drug administered and the parasite’s developmental stage. Promastigotes showed a marked increase in resistance when exposed to ML, with resistance ratios rising from a mean of 2.34 at round 5 to 20.60 by round 15 (**Figure 1**). In contrast, BEA-treated promastigotes exhibited more modest resistance development, reaching a mean ratio of 6.56 by round 15 (**Figure 1**). Statistical comparison between BEA and ML within each round of selection revealed no significant difference at round 5 (t(4) = -3.57, p > 0.01). However, ML resistance became significantly higher from round 10 onwards (t(4) = -5.57, p < 0.01; Generation 15: t(4) = -11.64, p < 0.01). Resistance development in amastigotes was generally lower for both drugs, with BEA displaying particularly stable resistance ratios across rounds of selection. Generation 5 differences remained non-significant (t(4) = -3.77, p > 0.01). However, by rounds 10 and 15, ML resistance was significantly elevated compared to BEA (t(4) = -23.25, p < 0.01; t(4) = -25.65, p < 0.01, respectively). The effects of BEA and ML on resistance development were consistent across different stages of the parasite (**Figure 1**). These findings underscore the greater potential for resistance development in promastigotes and the enhanced resistance observed in ML-treated intracellular amastigotes, whereas BEA may offer a more stable resistance profile across different developmental stages of *L. tropica*.

### 3.3 BEA increases intracellular calcium concentrations in *L. tropica*

**Figure 2:**
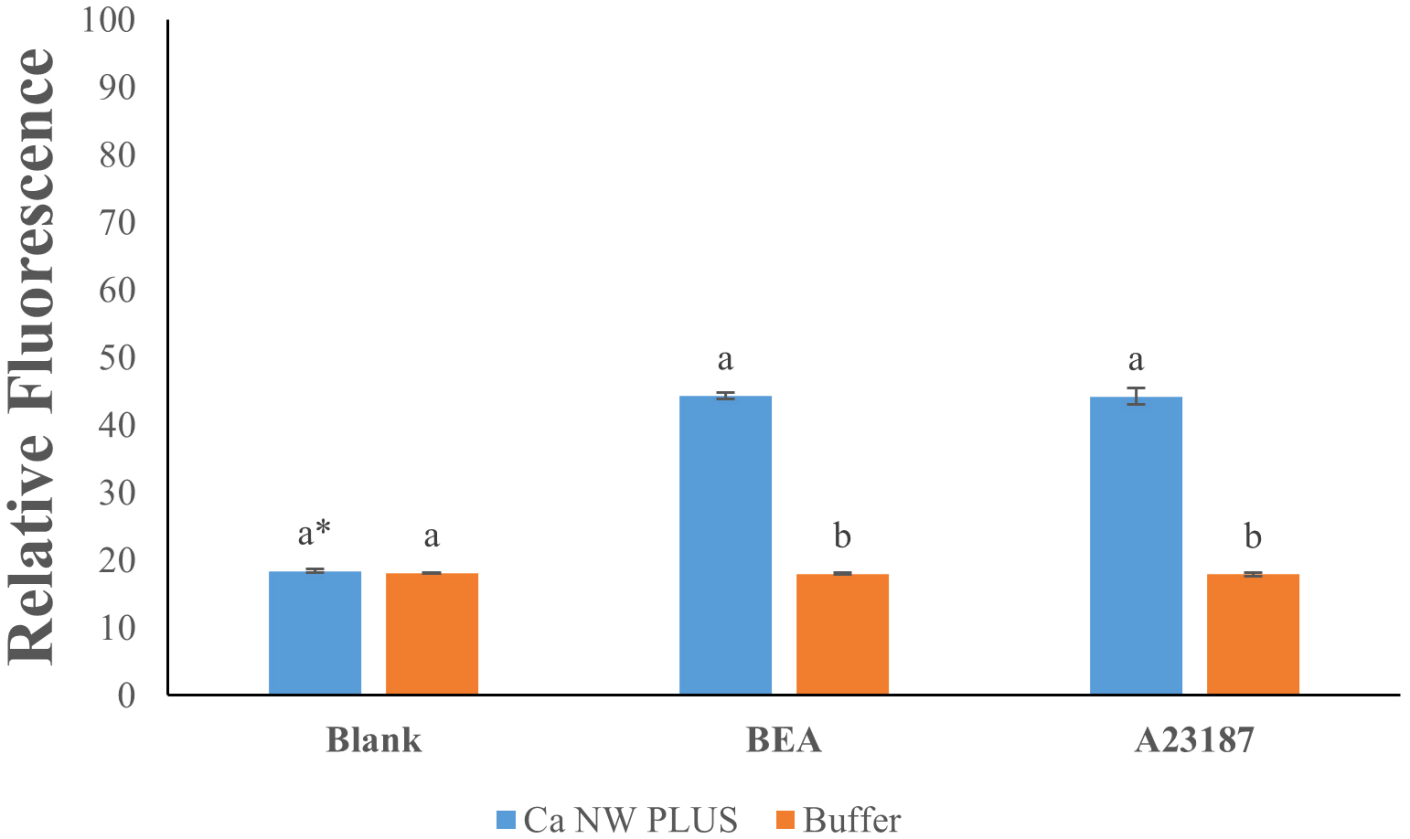
Effect of beauvericin (BEA) and A23187 on intracellular calcium levels in *Leishmania tropica.* * Bars with different letters indicate significant differences (p < 0.01).

Treatment of *L. tropica* with BEA elicited a significant elevation in intracellular calcium levels, evidenced by a 2.4-fold increase in fluorescence intensity compared to the untreated control (**Figure 2**) (df(2); F=1114; P ≤0.05). Approximately a similar 2.4-fold enhancement was observed with positive control A23187 (**Figure 2**), confirming the assay’s sensitivity in detecting intracellular calcium changes. Conversely, fluorescence readings in the buffer condition remained unchanged across all treatments, showing negligible variation (1-fold change) between the blank, BEA, and A23187. These findings substantiate the efficacy of BEA in significantly elevating intracellular calcium levels in *L. tropica*, demonstrating an effect comparable to that of the established calcium ionophore A23187.

### 3.4 Beauvericin-induced differential gene expression in *L. tropica*

**Figure 3:**
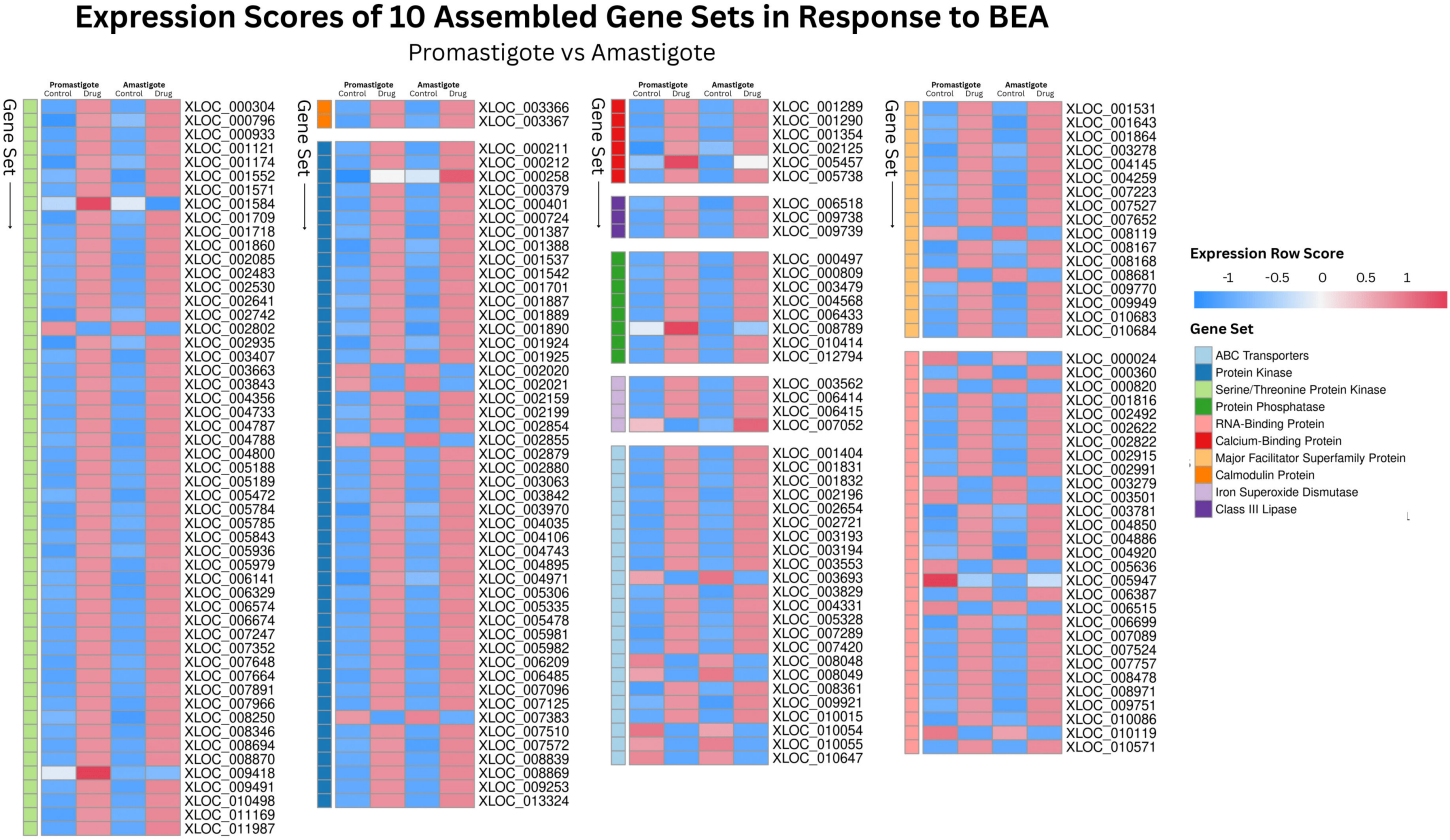
Heatmap of normalized transcript abundance for differentially expressed genes grouped by functional categories in *Leishmania tropica* promastigotes and intracellular amastigotes following BEA exposure. Expression data represent FPKM (Fragments Per Kilobase of transcript per Million mapped reads) values generated via the Tuxedo RNA-seq pipeline and visualized for genes that met the threshold of differential expression (FDR-adjusted p < 0.05) across four conditions: untreated promastigotes, BEA-treated promastigotes, untreated amastigotes, and BEA-treated amastigotes. Genes were assembled into ten functionally curated gene sets based on shared molecular roles. Color intensity reflects absolute expression levels (blue: low; red: high) across conditions, enabling visualization of condition- and stage-specific expression patterns in response to BEA.

**Figure 4:**
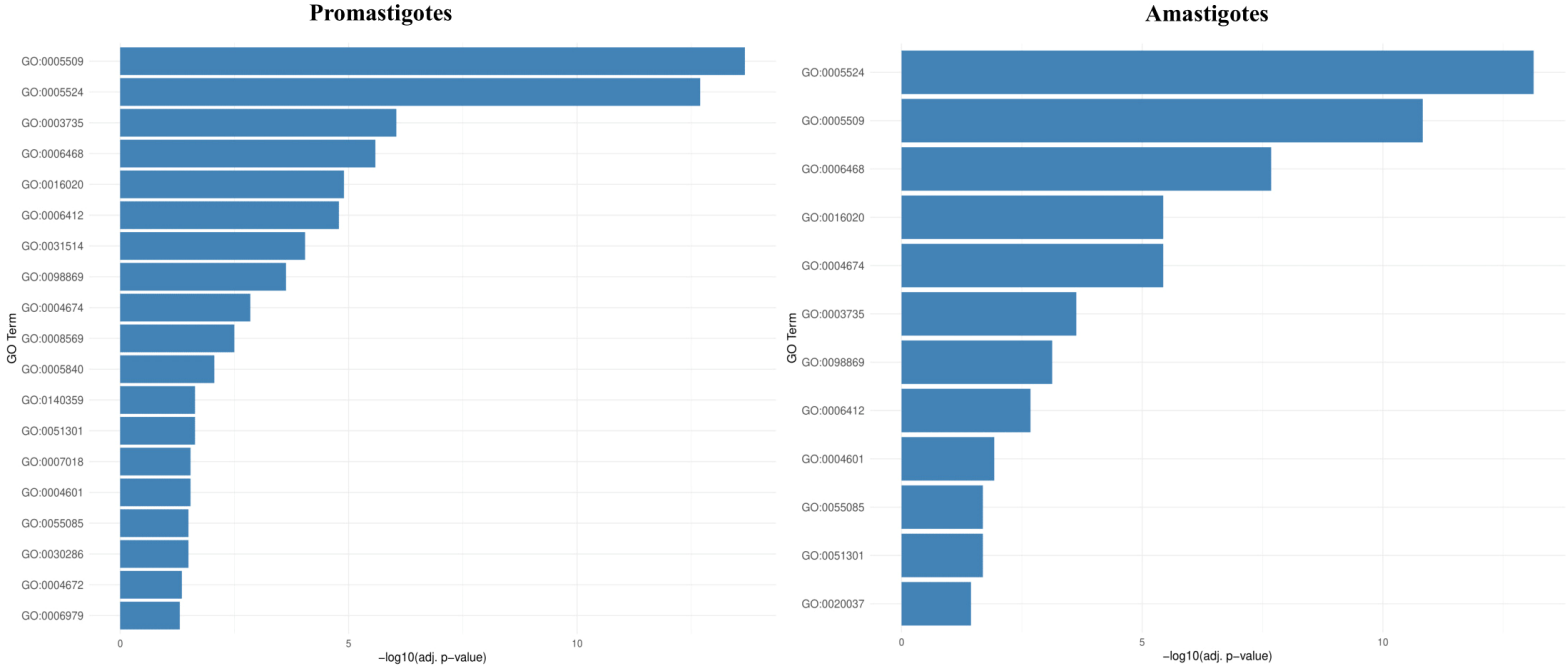
Over-representation analysis (ORA) reveals conserved and stage-specific functional enrichments following BEA treatment in *L. tropica*. Bar plots depict the top significantly enriched Gene Ontology (GO) terms among differentially expressed genes (DEGs) in promastigotes and intracellular amastigotes. Notable enrichments include transmembrane transport, protein phosphorylation, oxidative stress response, and calcium-binding functions, which were subsequently used to define biologically coherent gene sets for GSEA analysis.

RNA-Seq was employed to investigate BEA’s mode of action by evaluating changes in gene expression across both developmental stages of *L. tropica*. In the promastigote stage, 3,986 out of 8,653 genes (46%) were differentially expressed following BEA exposure, with 1,714 (43%) significantly upregulated and 2,272 (57%) significantly downregulated (**Supplementary Figure 1**). Similarly, in the intracellular amastigote stage, 3,509 out of 8,647 genes (40.5%) showed differential expression, with 1,551 (44.2%) upregulated and 1,958 (55.8%) downregulated (**Supplementary Figure 2**). These extensive changes suggest a widespread transcriptional response to BEA-induced membrane perturbation and major permeability changes at the membrane. In response, both promastigotes and intracellular amastigotes exhibited stage-independent overexpression of several specific gene sets in response to BEA (**Figure 3**). ORA of differentially expressed genes revealed that BEA treatment elicited enrichment of GO terms converging on a limited set of key biological processes central to parasite survival and adaptation. In both promastigotes and intracellular amastigotes, genes related to transmembrane transport, including ABC-type transporters and major facilitator superfamily proteins (GO:0055085, GO:0016020), were significantly overrepresented, suggesting the activation of membrane trafficking and detoxification pathways. Concurrently, GO terms associated with protein phosphorylation and kinase activity (GO:0006468, GO:0004672, GO:0004674) were strongly enriched, indicating broad engagement of signaling cascades likely involved in cellular reprogramming and stress adaptation. Additional enrichments in oxidative stress response (GO:0006979, GO:0098869) and calcium-binding activity (GO:0005509) further pointed towards mechanisms of redox balancing and calcium-dependent signaling triggered by BEA-induced ionic perturbations (**Figure 4**). These major biological themes, consistently emerging across developmental stages, guided the selection of gene sets for Gene Set Enrichment Analysis (GSEA). Specifically, genes grouped under calcium-binding proteins, transporters, kinases, oxidative stress enzymes, and translation-related categories were formalized into curated gene sets to systematically assess their coordinated regulation in response to BEA exposure.

### 3.5 *In vivo* therapeutic index of BEA

**Figure 5:**
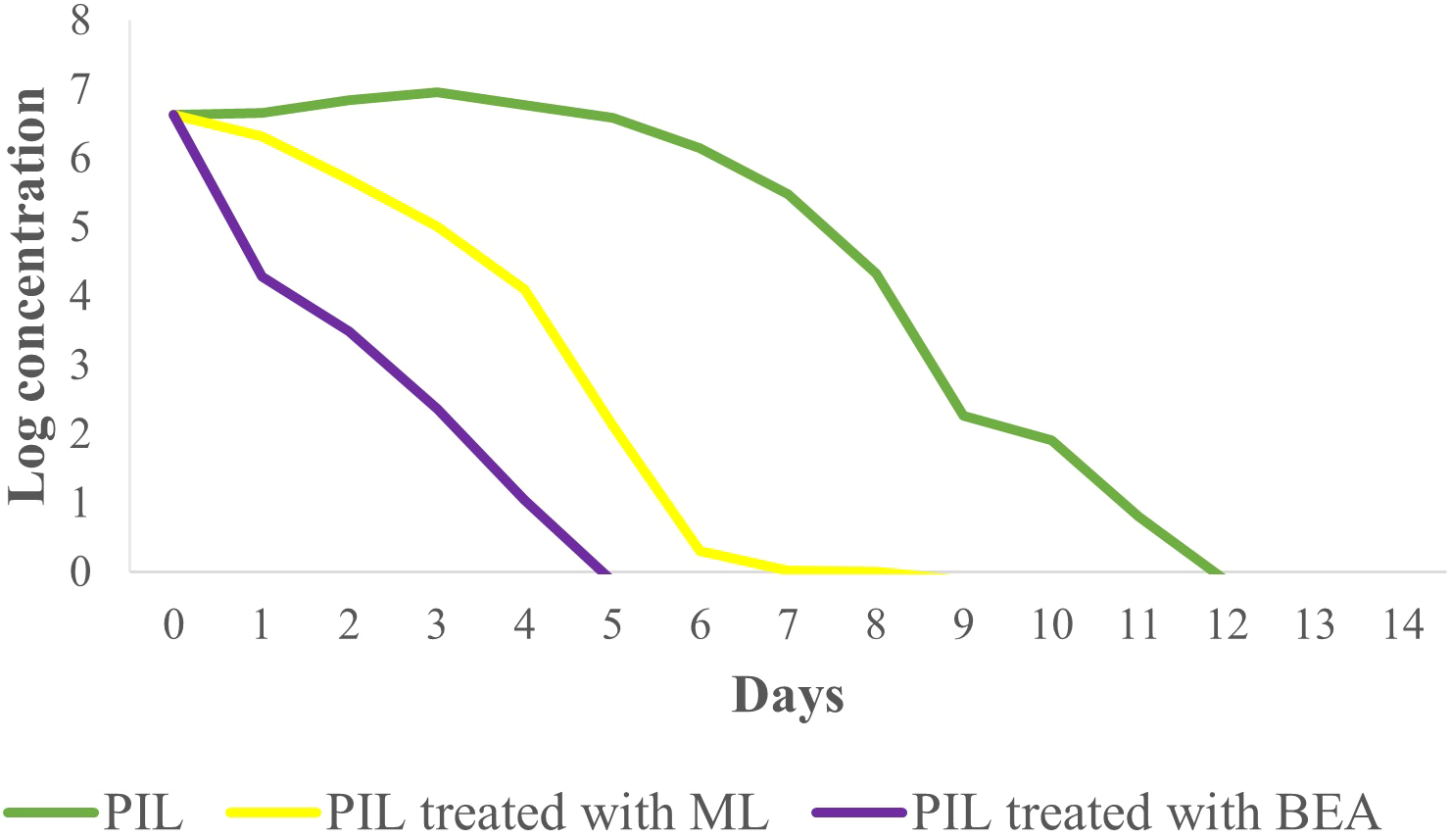
Effect of miltefosine (ML) and beauvericin (BEA) treatment on *Leishmania tropica* infection in *Galleria mellonella* with parasite burden measurement.

**Figure 6:**
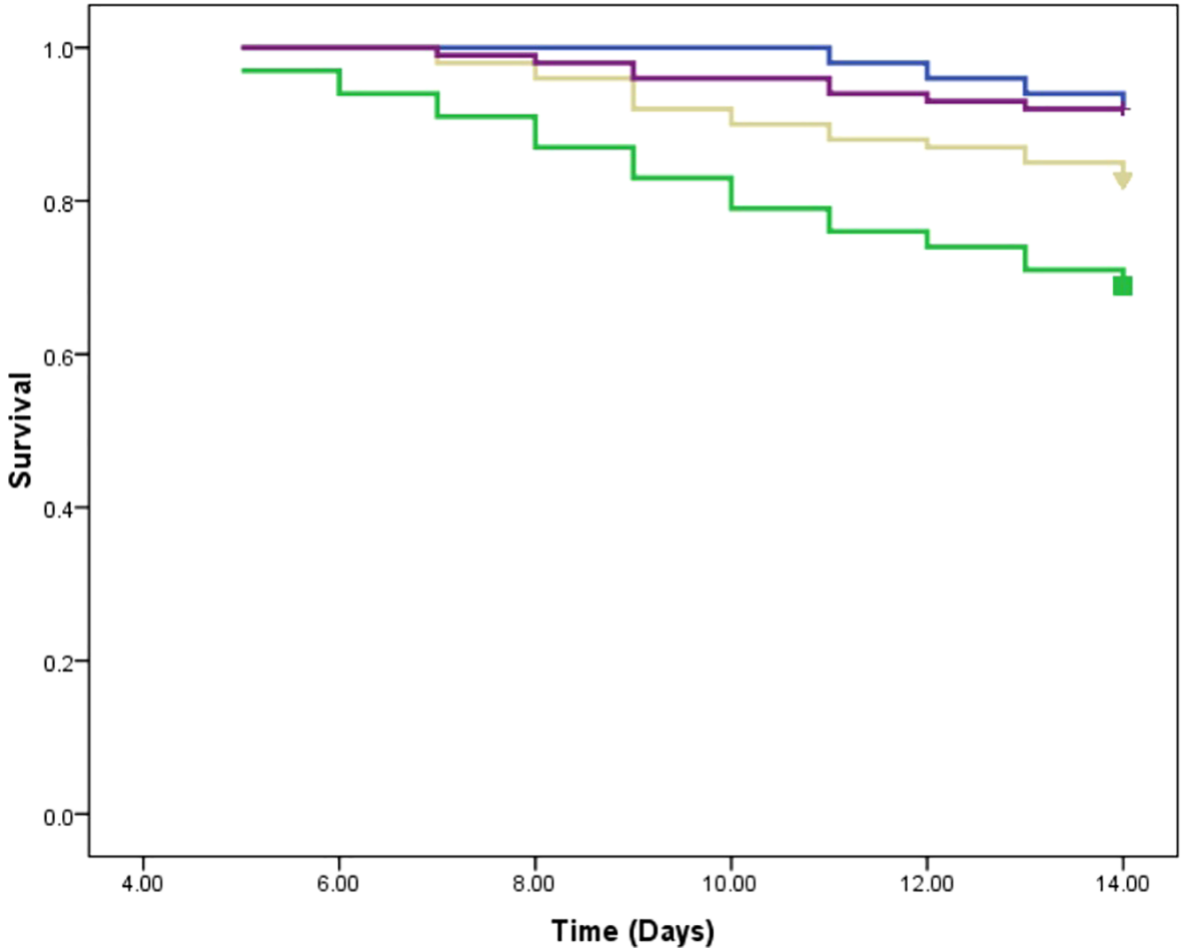
Survival analysis of *Galleria mellonella* larvae infected with *Leishmania tropica.* Blue: placebo; Green: promastigote infected larvae; Yellow: Promastigote infected larvae treated with miltefosine; Purple: Promastigote infected larvae treated with beauvericin.

**Figure 7:**
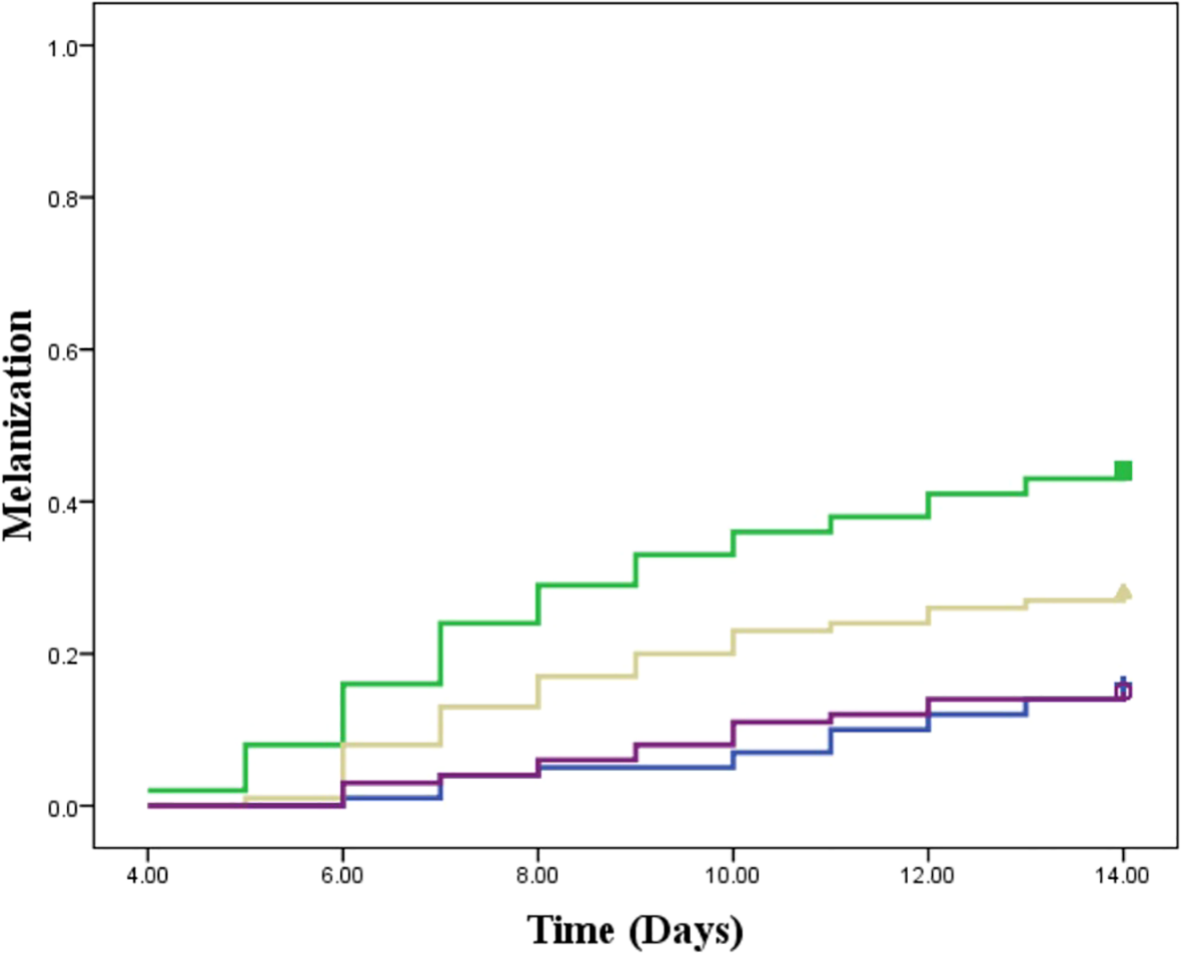
Melanization response in *Galleria mellonella* larvae infected with *Leishmania tropica.* Blue: placebo; Green: promastigote infected larvae; Yellow: Promastigote infected larvae treated with miltefosine; Purple: Promastigote infected larvae treated with beauvericin.

In this study, we assessed the therapeutic effects of different treatments on *L. tropica* burden within an insect model over a 14-day period by calculating the AUC for each group. The mean AUC values for each group were as follows: PIL exhibited a mean AUC of 57.30 ± 1.33 (mean ± SE), PIL treated with ML had a mean AUC of 29.85 ± 1.06, and the PIL treated with BEA showed the lowest mean AUC at 14.22 ± 0.86, indicating a significant reduction in *L. tropica* in the BEA-treated group compared to other groups. Statistical analysis using one-way ANOVA confirmed that these differences were highly significant (F(2, 57) = 188.71, p < 0.05), providing strong evidence that the treatments had markedly different effects on *L. tropica* burden. To further elucidate the specific differences between the groups, we conducted HSD test, which revealed that the mean AUC difference (95% cl) between the PIL and PIL treated with ML was 27.45 [22.50, 32.39] and a p-value of <0.001; between PIL and PIL treated with BEA was 43.08 [38.14, 48.03] and a p-value of <0.001; and between PIL treated with ML and PIL treated with BEA was 15.63 [10.69, 20.57] and a p-value of <0.001 (**Figure 5**). These results clearly demonstrate that the experimental drug BEA significantly reduced the burden of *L. tropica*. The mortality and melanization rates in the control group remained below 5% throughout the first 13 and 10 days post-exposure, respectively. A significant difference in mortality (**Figure 6**) and melanization (**Figure 7**) rates was observed between the control group and the PIL group. However, no statistically significant difference was found between the control group and the PIL group treated with BEA and ML, indicating that both MOX and ML demonstrated substantial therapeutic efficacy against *L. tropica*. Based on the chi-square analysis of disease burden, the groups can be ranked as follows: for mortality—PIL larvae (χ² = 18.172; df = 1; P < 0.05) > PIL treated with ML (χ² = 4.023; df = 1; P > 0.05) > PIL treated with BEA (χ² =0.004; df = 1; P > 0.05); and for melanization—PIL larvae (χ² = 21.111; df = 1; P < 0.05) > PIL treated with ML (χ² = 4.868; df = 1; P > 0.05) > PIL treated with BEA (χ² = 0.017; df = 1; P > 0.05).

## 4 Discussion

Leishmaniasis, particularly its cutaneous form caused by *L. tropica*, remains a significant global health challenge, particularly in regions with constrained healthcare infrastructure (Kim et al., 2025). Current therapies are hampered by inefficacy, severe side effects, and an alarming rise in drug resistance (Zhang et al., 2025). These challenges are compounded by the disproportionate burden on vulnerable populations (Akuffo et al., 2018). Despite its clinical relevance, *L. tropica* remains understudied, underscoring the need for new therapeutic strategies. BEA, a fungal secondary metabolite, has emerged as a promising candidate in the treatment of various parasitic infections, such as scabies and Chagas disease (Al Khoury et al., 2020; Luz et al., 1998). Notably, BEA has exhibited significant antileishmanial activity against *L. braziliensis*, which causes MCL, highlighting its potential as a broad-spectrum antileishmanial agent (Nascimento et al., 2012). Furthermore, recent investigations have indicated that BEA might partially counteract *L. tropica* resistance mechanisms by inhibiting the alternative ATP hydrolysis pathway (Al Khoury et al., 2024b).

In this study, BEA exhibited exceptional efficacy against all developmental stages of *L. tropica*, with a significantly lower IC_50_ value compared to ML, indicating its high potency as an antileishmanial agent. This lower IC_50_ indicates that BEA is considerably more potent, requiring much smaller concentrations to exert its antileishmanial effect. Such high potency offers important therapeutic advantages, including reduced toxicity risks, lower treatment costs, and a diminished likelihood of resistance development. These findings are consistent with previous reports showing that ML typically exhibits higher IC_50_ values (Vermeersch et al., 2009), with even greater values reported for older drugs like sodium stibogluconate. High-potency drugs, such as BEA, achieve therapeutic effects at lower concentrations and costs while minimizing resistance development (Oliveira et al., 2021). Moreover, such drugs are cost-effective, requiring lower doses, which is particularly advantageous in resource-limited settings, ultimately reducing the financial burden on healthcare systems and expanding treatment accessibility.

A key finding of this study is that BEA demonstrates equipotency across different developmental stages of *Leishmania*, a significant advantage over many existing leishmanicidal drugs that exhibit stage-specific effects (Costa et al., 2023; De Muylder et al., 2011). Identifying compounds with efficacy against all developmental stages is essential for reducing infection rates, decreasing parasitemia, and improving survival outcomes. Although this concept has been well established in malaria drug discovery (Williams and Le Roch, 2019), it is equally relevant to *Leishmania*, where multi-stage active compounds could offer similar therapeutic advantages. Although the promastigote and amastigote stages occur in different hosts—the sandfly vector and the human host, respectively—equipotency is crucial for comprehensive disease management. While the amastigote stage remains the primary therapeutic target within the mammalian host, BEA’s ability to target promastigotes has significant implications for preventing reinfection. In endemic regions, individuals recovering from leishmaniasis frequently encounter subsequent bites from sandflies carrying promastigotes. If present in the bloodstream, BEA may impair the viability of newly introduced promastigotes before they are internalized by macrophages and differentiate into amastigotes, potentially reducing the establishment of secondary infections. While this possible dual action requires experimental validation, it raises the prospect of post-treatment prophylaxis, which could help reduce relapse risk and improve long-term disease management in high-transmission settings (Ponte-Sucre et al., 2017). However, while this concept of post-treatment prophylaxis is intriguing, it should be considered with caution. Persistent sub-curative drug levels may paradoxically create a selective environment that favors the emergence of tolerant or resistant parasites. As such, the potential for dual-stage action remains hypothetical at this stage, and its clinical relevance requires further experimental validation to balance potential prophylactic benefits against the risk of drug resistance development.

Another notable observation is the relatively high cytotoxic effect of BEA on macrophage-like cells compared to ML. However, it’s important to emphasize that the concentration required to induce cytotoxicity was approximately ten times higher than the effective dose needed to eliminate the parasite. Although this therapeutic index is considered acceptable, it remains moderate and indicates that BEA’s safety margin may not be optimal, particularly in systemic or long-term applications. This underscores the necessity for a cautious interpretation of its selectivity and highlights the potential need for formulation strategies or targeted delivery approaches to reduce host cell exposure and enhance parasite-specific activity. This favorable therapeutic index is particularly critical in infections like leishmaniasis, where the target cells are host-derived (Sheikh et al., 2024; Novais et al., 2021). BEA has demonstrated significant therapeutic potential across diverse applications, including anticancer (Heilos et al., 2017), antifungal (Rana et al., 2022), and antiviral (Al Khoury et al., 2022b). Its safety profile has been extensively evaluated in animal models, showing good tolerance with no significant systemic toxicity, even after multiple doses (Heilos et al., 2017). Despite BEA accumulation in tissues such as tumor tissues and fat, it did not induce pathological changes or adverse effects in major organs, including the liver, kidneys, and lungs (Heilos et al., 2017). Furthermore, *in vitro* and *in vivo* studies by Maranghi et al. (2018) confirmed BEA’s safety, reporting low genotoxicity and manageable immunotoxic effects at therapeutic doses, with a No Observed Adverse Effect Level (NOAEL) of 1 mg/kg in female mice and 0.1 mg/kg in male mice. Pharmacokinetic studies also highlight BEA’s metabolic stability, high plasma protein binding, and extended half-life, contributing to its favorable pharmacokinetic profile (Yuan et al., 2022). Additionally, BEA demonstrated the capacity to permeate biological barriers, including the skin, without inducing significant cytotoxicity (Taevernier et al., 2016). This property suggests the potential for topical administration, which may offer important pharmacological advantages in the context of CL. Localized delivery directly to the site of infection could limit systemic exposure, thereby mitigating concerns associated with the moderate therapeutic index observed *in vitro*. Furthermore, topical formulations are particularly relevant for CL, where the disease is confined to the skin and where treatment strategies that minimize host toxicity while maintaining antiparasitic efficacy are especially desirable. Although this possibility remains to be formally investigated, future studies should consider the evaluation of BEA as a topical therapeutic candidate, both to enhance selectivity and to improve treatment accessibility in resource-limited settings. Collectively, these findings suggest that BEA possesses a favorable safety profile and pharmacokinetic characteristics, reinforcing its potential as a viable therapeutic agent.

A hallmark of *Leishmania* spp. is their adaptability to environmental changes, such as pharmacological fluctuations within human host cells, which significantly contributes to drug resistance (Bussotti et al., 2018). In this study, resistance development was more pronounced in promastigotes than in amastigotes, especially when exposed to ML. This difference can be attributed to the direct exposure of promastigotes to higher drug concentrations in the extracellular environment, resulting in greater selective pressure and faster resistance development. In contrast, amastigotes, residing within macrophages, encounter the drug in a more protected intracellular setting, which likely reduces the effective concentration and slows resistance acquisition. These findings align with previous studies by Hendrickx et al. (2012), emphasizing the need to study both developmental stages to comprehensively understand resistance mechanisms. A significant observation in this study is that BEA exhibited substantially lower resistance acquisition compared to ML. This outcome is consistent with previous reports of rapid ML resistance development in species such as *L. donovani* (Pérez-Victoria et al., 2003), *L. braziliensis* (Obonaga et al., 2014), *L. major* (Turner et al., 2015), *L. amazonensis* (Trinconi et al., 2016), and *L. infantum* (Carnielli et al., 2019). As a hexadepsipeptide ionophore, BEA increases membrane permeability by facilitating the transmembrane flux of calcium and other cations, thereby disrupting intracellular ionic homeostasis (Wang and Xu, 2012). This mechanism is less susceptible to single-point mutations, which are often the basis of resistance to drugs with specific targets, such as ML. For example, (Cojean et al., 2012) identified a single-nucleotide polymorphism, L832F, in the ML-transporter gene (Ldmt), which serves as a marker for ML resistance. In contrast, BEA, lacking a single, specific target, exerts a broader, more disruptive influence on cellular function, likely contributing to its slower resistance development.

Elucidating a drug’s mechanism of action is crucial for improving efficacy and overcoming resistance in *Leishmania* treatment. BEA, a hexadepsipeptide ionophore, disrupts ion homeostasis by increasing membrane permeability to calcium ions (Mallebrera et al., 2018). This causes a rise in intracellular calcium, triggering downstream effects such as reactive oxygen species (ROS) production and apoptosis (Mallebrera et al., 2018). Intracellular calcium stability is essential for *Leishmania*, as it regulates vital processes, including macrophage invasion, flagellar motility, and mitochondrial metabolism (Kashif et al., 2017). The high intracellular calcium levels observed in BEA-treated *L. tropica* further confirm that BEA acts as a potent calcium mobilizer, consistent with its ionophoric mechanism of action. By permeabilizing the plasma membrane, BEA disrupts the parasite’s ability to maintain ionic gradients, resulting in a rapid and uncontrolled influx of calcium ions. Membrane permeabilization and calcium dysregulation are thus established as the central initiating events in BEA’s antileishmanial activity, setting in motion a cascade of compensatory and maladaptive responses that ultimately lead to parasite demise. To investigate the cellular response to this calcium-driven ionic disruption, we performed transcriptome-wide RNA sequencing to capture the downstream molecular adaptations following BEA-induced membrane disruption.

RNA-Seq analysis revealed significant transcriptional remodeling in *L. tropica*. Consistent with the elevation in intracellular calcium, RNA-Seq analysis revealed upregulation of six calcium-binding protein genes and three calmodulin-encoding genes across both developmental stages (**Table 2**; **Figure 3**). This response aligns with the known role of calcium-binding proteins and calmodulin in regulating stress signaling pathways critical for parasite survival (Miranda et al., 2014; Mukhopadhyay and Dey, 2017). Elevated calcium levels are further implicated in the activation of apoptosis pathways, reinforcing the cytotoxic role of BEA-induced ionic imbalance (Tong et al., 2016). In parallel, we detected marked upregulation of several protein kinase genes, including serine/threonine kinases (**Table 2** **;** **Figure 3**), likely reflecting calcium/calmodulin-dependent activation of stress-responsive signaling cascades (Martín, 2023; Naula et al., 2005).

**Table 2:** Representation of the differentially expressed gene sets in promastigotes and intracellular amastigotes with their GO IDs, the number of genes within the gene set, the number of differentially expressed genes within each gene set, and their functions (*p* < 0.05) following BEA exposure.

Gene set	GO ID	Function	Number of genes per gene set	Promastigotes					Intracellular amastigotes				
				Number of DEG	Expression level	*p*-value	Log2FC	ES	Number of DEG	Expression level	*p*-value	Log2 FC	ES
ABC transporters	P:GO:0055085; F:GO:0005524; F:GO:0140359; C:GO:0016020	P:transmembrane transport; F:ATP binding; F:ABC-type transporter activity; C:membrane	35	25	Upregulated	0.003	0.407	0.437	23	Upregulated	0.004	0.407	0.471
Protein kinase	P:GO:0006468; F:GO:0004672; F:GO:0005524	P:protein phosphorylation; F:protein kinase activity; F:ATP binding	69	49	Upregulated	1.659e-7	0.690	0.530	48	Upregulated	6.786e-8	0.704	0.535
Serine/Threonine Protein kinase	P:GO:0006468;F:GO:0004674;F:GO:0005524	P:protein phosphorylation; F:protein serine/threonine kinase activity; F:ATP binding	80	53	Upregulated	9.027e-9	0.747	0.535	53	Upregulated	2.878e-8	0.733	0.531
RNA-binding protein	F:GO:0003723	F:RNA binding	80	36	Upregulated	0.0007	0.477	0.439	29	Upregulated	0.003	0.431	0.448
Calcium binding protein	F:GO:0005509; C:GO:0016020	F:calcium ion binding; C:membrane	6	6	Upregulated	0.002	0.431	0.764	6	Upregulated	0.004	0.407	0.756
Major facilitator superfamily protein	P:GO:0055085; F:GO:0022857; C:GO:0016020	P:transmembrane transport; F:transmembrane transporter activity; C:membrane	33	17	Upregulated	0.001	0.455	0.563	17	Upregulated	0.0009	0.477	0.567
Calmodulin protein	P:GO:0006468; F:GO:0004672; F:GO:0005509; F:GO:0005524	P:protein phosphorylation; F:protein kinase activity; F:calcium ion binding; F:ATP binding	3	3	Upregulated	0.002	0.431	0.896	2	Upregulated	0.019	0.352	0.889
Iron superoxide dismutase	P:GO:0019430; F:GO:0004784; F:GO:0046872	P:removal of superoxide radicals; F:superoxide dismutase activity; F:metal ion binding	4	4	Upregulated	0.0003	0.498	0.895	4	Upregulated	0.0001	0.538	0.904
Class III lipase	P:GO:0006629; F:GO:0046872	P:lipid metabolic process; F:metal ion binding	3	3	Upregulated	0.002	0.431	0.907	3	Upregulated	0.001	0.455	0.909

The concurrent overexpression of ABC transporter genes (**Table 2**; **Figure 3**) suggests an attempt to extrude toxic metabolites or compensate for membrane damage, although BEA’s ability to inhibit ABC transporters may counteract this protective mechanism (Vanaerschot et al., 2014; Al Khoury et al., 2024b). Additionally, we observed significant induction of RNA-binding proteins (RBPs), which are central regulators of post-transcriptional adaptation under stress (**Table 2**; **Figure 3**) (Karamysheva et al., 2020; Nocua et al., 2017). Upregulation of RBPs likely reflects a strategy to preserve mRNA integrity and sustain critical gene expression programs during cytotoxic stress (Yamoah et al., 2023).

Other transcriptional adaptations included upregulation of major facilitator superfamily (MFS) transporter genes (**Table 2**; **Figure 3**), potentially reflecting efforts to regulate intracellular iron homeostasis and mitigate oxidative stress (Landfear, 2019; Kelly et al., 2021; Sarkar et al., 2019). The observed induction of class III lipase genes (**Table 2**; **Figure 3**) may represent a detoxification mechanism, as lipases have been implicated in ester bond hydrolysis of xenobiotic compounds. Finally, the significant upregulation of iron superoxide dismutase (SOD) genes (**Table 2**; **Figure 3**) underscores a defensive response to ROS-mediated oxidative stress, in line with previous studies highlighting SOD’s role in promoting *Leishmania* survival under oxidative challenge (Plewes et al., 2003; Huynh and Andrews, 2008; Veronica et al., 2019).

Given the promising cellular effects of BEA, we extended our investigation to assess its therapeutic potential *in vivo*. This study provides the first *in vivo* evidence of BEA’s potent antileishmanial activity, demonstrating a significant reduction in parasite burden and improved host survival. While *Galleria mellonella* is not a conventional model for *Leishmania*, the use of invertebrate models in parasitic infection research is well-established. *Drosophila melanogaster* and *Caenorhabditis elegans* have been successfully used to study intracellular parasites such as *Nematocida parisii*, *Octosporea muscaedomesticae*, and *L. amazonensis* (Nainu et al., 2022; Troemel et al., 2008; Roxström-Lindquist et al., 2004). These precedents support the use of *G. mellonella* as a cost-effective and ethically viable platform to assess drug efficacy before transitioning to mammalian models.

The reduction in parasite burden in untreated larvae suggests that *Galleria*’s immune system partially controls infection. Hemocytes, analogous to mammalian phagocytes, mediate pathogen elimination through phagocytosis and melanization (Ménard et al., 2021). The intensified melanization observed in infected larvae (**Figure 7**) suggests an enhanced immune response to *L. tropica*, consistent with Tomiotto-Pellissier et al. (2016). The *ex vivo* studies showed that *G. mellonella* hemocytes can eliminate *L. braziliensis* amastigotes. Our study provides the first *in vivo* evidence of *Galleria*’s immune responsiveness to *Leishmania*. However, persistent parasitemia in untreated larvae highlights the need for pharmacological intervention to achieve effective parasite clearance. Critically, BEA-treated larvae exhibited the most pronounced reduction in parasite burden, far exceeding the effects observed in untreated and ML-treated groups (**Figure 5**).

Beyond its direct antiparasitic effects, BEA treatment also improved host survival rates, suggesting an additional immunomodulatory role (**Figure 6**). BEA has been shown to activate Toll-like receptor 4 (TLR4) signaling pathways, leading to the production of pro-inflammatory cytokines such as interleukin-12 (IL-12) and interferon-beta (IFN-β) (Yang et al., 2022). These cytokines are crucial for activating macrophage-mediated parasite clearance in mammals and may play a comparable role in *G. mellonella*’s immune system. Additionally, BEA has been reported to modulate antimicrobial peptide (AMP) production, potentially enhancing hemocyte-mediated pathogen elimination (Patiño-Márquez et al., 2018). This suggests that BEA’s efficacy extends beyond direct cytotoxicity to include immune-enhancing effects, further strengthening its potential as a therapeutic candidate.

In contrast, ML treatment resulted in only moderate parasite clearance, with relatively high mortality in infected larvae (**Figure 6**). The limited efficacy of ML may be attributed to several factors. ML exerts its antiparasitic effects by integrating into parasite membranes and disrupting lipid metabolism (Dorlo et al., 2014); however, the lipid-rich tissues of *Galleria mellonella* may sequester ML, reducing its bioavailability. Additionally, ML’s slow pharmacokinetics and prolonged half-life may further limit its efficacy in this short-term infection model (Palić et al., 2020). This aligns with previous reports indicating that ML’s therapeutic effectiveness varies significantly depending on geographical region, host factors, and parasite strain diversity (Dorlo et al., 2014; Fernández et al., 2014). These findings highlight BEA’s superior efficacy compared to ML, further supporting its therapeutic potential.

## 5 Conclusion

This study provides compelling evidence of the antileishmanial potential of BEA against *L. tropica*, demonstrating its efficacy across all developmental stages, including both promastigotes and intracellular amastigotes. Compared to the current treatment standard, ML, BEA exhibited superior potency, equipotency across stages, and a lower tendency for resistance development, highlighting its potential as a more robust therapeutic option. Furthermore, our RNA-Seq analysis revealed that BEA disrupts critical molecular pathways in *L. tropica*, including calcium homeostasis, lipid metabolism, and stress response mechanisms, contributing to its multifaceted mechanism of action. The use of the *G. mellonella* model provided valuable *in vivo* insights into BEA’s therapeutic index, suggesting that BEA not only exerts direct antiparasitic effects but may also enhance host immune responses. This dual action positions BEA as a promising candidate for developing novel antileishmanial therapies, particularly in resource-limited settings where cost-effective and efficient treatments are urgently needed. Given the challenges of drug resistance and the limited efficacy of current treatments, our findings underscore the potential of BEA as a viable alternative or adjunctive therapy for leishmaniasis. Future research should focus on validating BEA’s efficacy in mammalian models, exploring its immunomodulatory properties further, and investigating its potential in combination therapies to enhance treatment outcomes. Collectively, this study lays a strong foundation for the continued exploration of BEA as a novel therapeutic agent in the fight against leishmaniasis.

## Data Availability

The datasets generated and/or analyzed during the current study are available in the NCBI Sequence Read Archive (SRA) under BioProject accession number PRJNA1273512. All associated raw reads and metadata can be accessed through this BioProject.
