# Supplementary material for: Evaluation of Beauvericin’s activity and mode of action against all life stages of L. tropica for cutaneous Leishmaniasis therapy
Source: Front Cell Infect Microbiol. 2025 Jun 10;15:1599766. doi: 10.3389/fcimb.2025.1599766 (PMC12185981; doi:10.3389/fcimb.2025.1599766)
Supplement: Supplementary file 1 [file Table1.docx]

**Supplementary note 1: Gene families**

Interestingly, the calmodulin gene set gained distinction. This gene set consisted of six genes encoding proteins affiliated with the following Gene Ontology (GO) terms: GO:0006468 (protein phosphorylation), GO:0004672 (protein kinase activity), GO:0005509 (calcium ion binding), GO:0005524 (ATP binding) (Table 1). Furthermore, six genes corresponding to the calcium-binding protein gene set were identified by GO:0005509 (calcium ion binding) and GO:0016020 (membrane) (Table 1). Additionally, the ATP-binding cassette (ABC) transporters gene set, encompassing 35 genes annotated with GO:0055085 (transmembrane transport), GO:0005524 (ATP binding), GO:0140359 (ABC-type transporter activity), GO:0016020 (membrane), was associated with drug resistance (Table 1). The protein kinase, serine/threonine protein kinase, and the phosphatase protein gene sets encompassing 69, 80, and 13 genes defined by GO:0006468 (protein phosphorylation), GO:0004674 (protein serine/threonine kinase activity), GO:0004672 (protein kinase activity), GO:0005524 (ATP binding) and GO:0017018 (myosin phosphatase activity) respectively, are characterized by their involvement in regulating the parasite’s life cycle (Table 1). To ensure transmembrane transport through iron efflux, the major facilitator superfamily (MFS) gene set, encompassing 33 genes (Table 1) annotated with GO:0055085 (transmembrane transport), GO:0022857 (transmembrane transporter activity), GO:0016020 (membrane) was constructed. The primary gene regulators, RNA-binding proteins, constitute a set of 80 genes (Table 1) annotated with GO:0003723 (RNA binding). The iron superoxide dismutase gene set, comprising four genes (Table 1) identified by GO:0019430 (removal of superoxide radicals), GO:0004784 (superoxide dismutase activity), and GO:0046872 (metal ion binding), are involved in the pathogenicity of the parasite. As part of *Leishmania*’s exoproteome, the class III lipase gene set involved three genes (Table 1) identified by GO:0006629 (lipid metabolic process) and GO:0046872 (metal ion binding).

**Supplementary Figure 1:** Differential gene expression profiles in *Leishmania tropica* promastigotes following beauvericin (BEA) exposure.


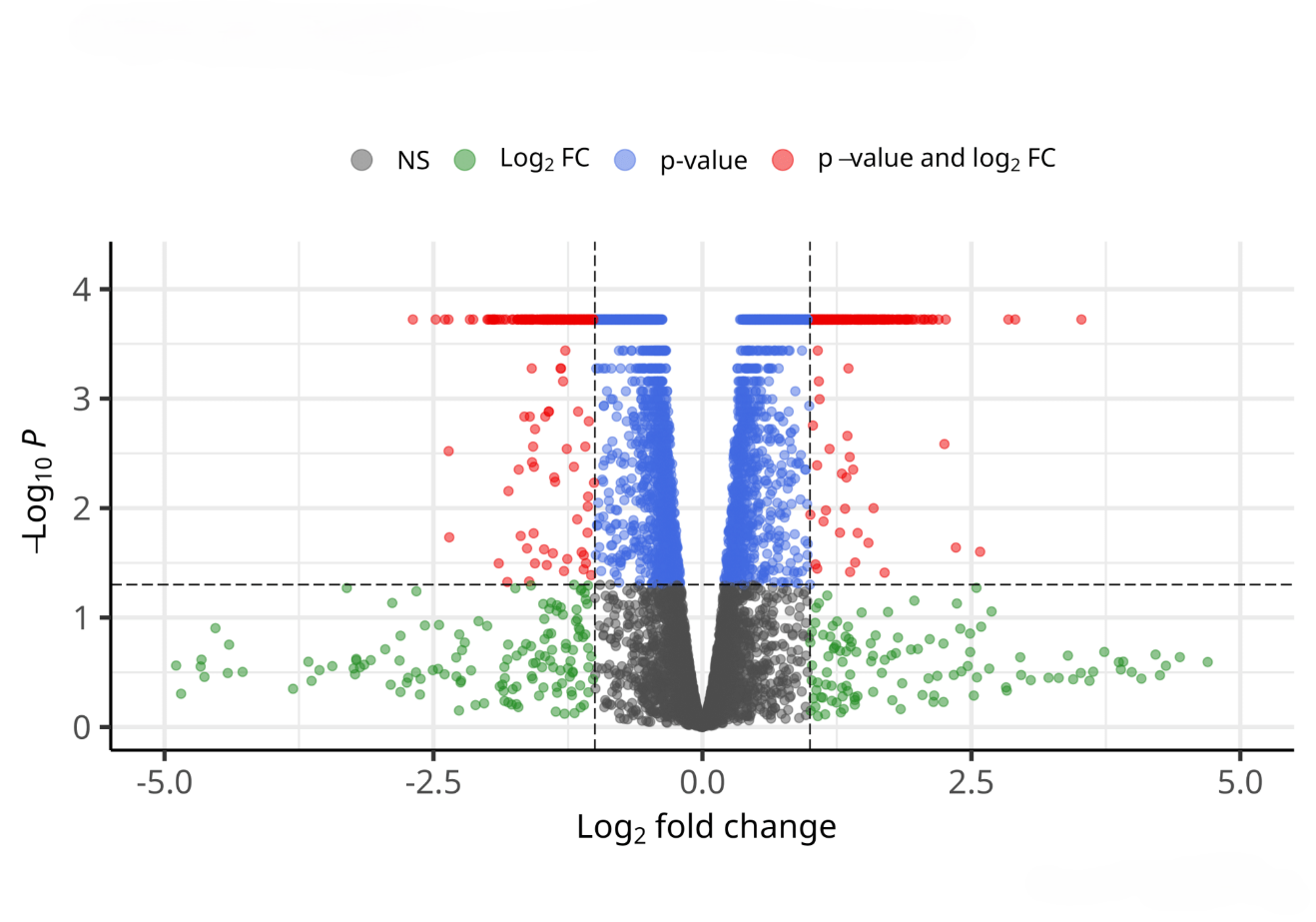
Volcano plots illustrate the transcriptomic changes between untreated controls and BEA-treated *L. tropica* promastigotes. Each point represents a gene. The x-axis denotes the log₂ fold change in gene expression, and the y-axis indicates the –log₁₀ of the p-value. Genes meeting the threshold for both statistical significance (p < 0.05) and substantial expression change (|log₂FC| > 1) are highlighted in red. Genes with only significant fold change (green) or only p-value < 0.05 (blue) are also marked. Non-significant genes are shown in gray.

**Supplementary Figure 2:** Differential gene expression profiles in *Leishmania tropica* amastigotes following beauvericin (BEA) exposure.


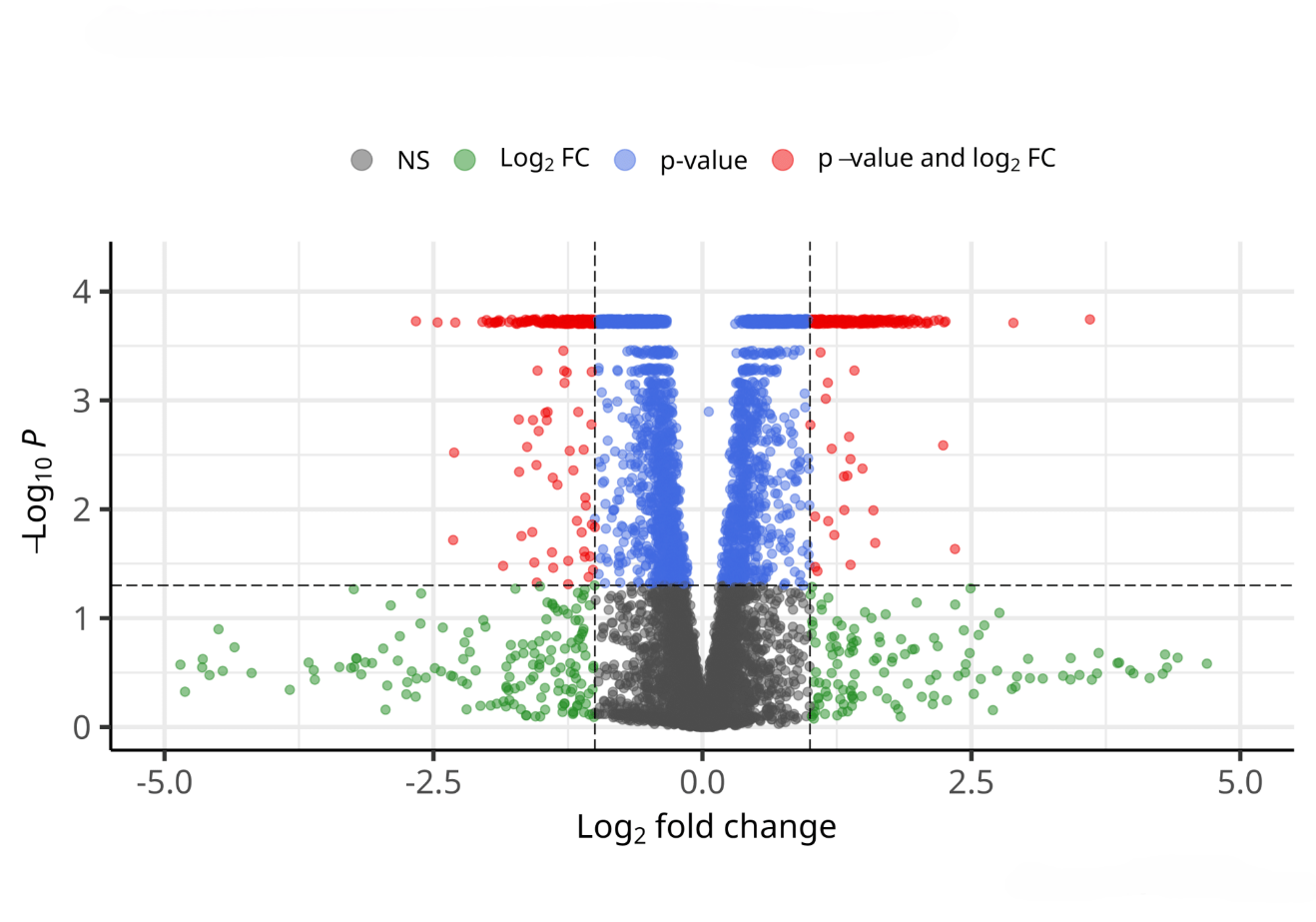


Volcano plots illustrate the transcriptomic changes between untreated controls and BEA-treated *L. tropica* amastigotes. Each point represents a gene. The x-axis denotes the log₂ fold change in gene expression, and the y-axis indicates the –log₁₀ of the p-value. Genes meeting the threshold for both statistical significance (p < 0.05) and substantial expression change (|log₂FC| > 1) are highlighted in red. Genes with only significant fold change (green) or only p-value < 0.05 (blue) are also marked. Non-significant genes are shown in gray.
